# ATTITUDES TOWARD AGING AND SUBJECTIVE COGNITIVE COMPLAINTS: DOES DEPRESSION MODERATE THEIR RELATIONSHIP?

**DOI:** 10.1093/geroni/igae098.0026

**Published:** 2024-12-31

**Authors:** Fatema Colombowala, Emily Bower

**Affiliations:** Pacific University, Hillsboro, Oregon, United States; Pacific University, Hillsboro, Oregon, United States

## Abstract

Subjective cognitive decline (SCD) is a risk factor for Alzheimer’s disease and related dementias and affects a significant portion of the population, with prevalence ranging from 12.3% to 57% of older adults. Although some people with SCD will later develop dementia, others will not, suggesting that factors other than objective cognitive ability impact subjective cognition. Attitudes towards aging (ATA) may influence how individuals interpret age-related cognitive changes, potentially leading to heightened subjective cognitive complaints (SCC) among those who hold more negative attitudes toward aging. We investigated the relationship between ATA and SCC in a sample of older adults with SCD. We hypothesized negative attitudes would be associated with more SCC. Additionally, we examined if depression moderated the relationship between ATA and SCC, predicting that more severe depression would strengthen the relationship. There is a scarcity of studies that directly compare the strength of association between SCC and various outcomes such as depression and ATA. Preliminary analyses included 51 participants, predominantly female (72.5%), with an average age of 72 (SD = 8.5) who had SCD. They completed validated measures of SCC, aging attitudes (ATA), and depression as part of a larger study. Hypotheses were tested using linear regression models. We found more negative attitudes on the ATA psychosocial loss subscale were associated with higher SCC (B = 26.50, p =.007). Additionally, a diagnosis of depression moderated the relationship by strengthening it (B = 35.80, p =.0096). Findings suggest intervening to reduce depression and negative ATA could reduce SCC burden.

